# Myelofibrosis-Associated Lymphoproliferative Disease: Retrospective Study of 16 Cases and Literature Review

**DOI:** 10.1155/2009/179847

**Published:** 2009-11-25

**Authors:** A. Etienne, B. Gruson, D. Chatelain, R. Garidi, B. Royer, H. Sevestre, J. P. Marolleau, G. Damaj

**Affiliations:** ^1^Department of Clinical Hematology, Centre Hospitalier Universitaire, University Hospital of Amiens, 1 Place Victor Pauchet, 80000 Amiens, France; ^2^Department of Clinical Pathology, University Hospital of Amiens, 1 Place Victor Pauchet, 80000 Amiens, France

## Abstract

*Background*. To better describe the clinical, biological, and the outcome of non-Hodgkin's lymphoma (NHL) with, at the initial presentation, bone marrow fibrosis (MF). *Patients and Methods*. From January 2001 to January 2007, 16 eligible patients with NHL and MF were retrieved from the Pathology Department of the University hospital of Amiens. Median age of patients was 62 years (range 16–74) with a sex ratio male/female of 3. *Results*. MF is associated with all types of lymphoma predominantly with B-cell phenotype and it seems to be more associated with low-grade NHL. B-symptoms are more frequent at diagnosis and more patients presented with an elevated LDH level. JAK-2 was negative in the 10 patients analysed. Two patients presented with features of primary MF with no evidence of lymphoma. Overall response rate was 94% after the first line of therapy with regression or improvement of MF. Relapse occurred in 8 patients (47%) with recurrence of MF in all of them. After a median follow-up of 42 months, 12 patients were alive with an overall survival rate for the entire group of 75%. *Conclusions*. MF-associated NHL is a rare manifestation which may be associated with all types of NHL and its presence does not seem to confer a poor prognosis. A search for lymphoproliferation should be considered when the cause of MF is not apparent.

## 1. Introduction

Myelofibrosis (MF) is characterized by the accumulation of reticulin and collagen fibers and the proliferation of fibroblastic mesenchymal cells in the bone marrow in association with increased neoangiogenesis and circulating levels of particular cytokines such as TGF-*β*1, PDGF, and FGF-*β* [1]. 

MF can be classified into two categories: primary idiopathic (IMF) and secondary process occurring at the onset or in the course of the disease. Chronic myeloproliferative neoplasms (polycythemia vera, essential thrombocythemia, or chronic myeloid leukemia) represent one of the frequent causes of secondary MF followed by autoimmune myelofibrosis or other nonhematologic disorders [2]. The association between MF and lymphoproliferative disease is a very rare event and is not well described except in multiple myeloma [3] and hairy cell leukemia. Few cases reported the association between MF and lymphoproliferation [4–16]. However, the true incidence of this association, the clinical characteristics, and the prognosis of the patients are not known. 

In this report, we review literature and describe the clinical characteristics and outcome of 16 patients with lymphoproliferative disorders associated, at diagnosis, with MF.

## 2. Patients and Methods

### 2.1. Patients

From January 2001 to January 2007, 25 previously untreated patients with the diagnosis of lymphoproliferative disease and bone marrow MF on initial bone marrow (BM) evaluation were retrieved from the archives of the Department of Pathology of the University Hospital of Amiens. To be eligible for the study, patients with B- or T-cell lymphoproliferative diseases should have MF based on the initial bone marrow histology examination.

### 2.2. Diagnostic and Staging Procedures

Medical files and histological blades of all patients were re-examined. Patients with the diagnosis of hairy cell leukemia were excluded because of its frequent and known association with BM fibrosis. The diagnostic work-up and staging procedures on presentation included patient history and complete physical examination, full blood cell count, serum lactate dehydrogenase (LDH), *β*2-microglobulin, liver enzymes, alkaline phosphatase and creatinine levels, albumin level, chest X-ray, and computed tomography (CT) scan of the chest, abdomen and the pelvis. Patients were staged according to the Ann Arbor staging system. The mutational status for JAK2^V617F^ was determined retrospectively in 10 patients using a PCR assay as described [17].

### 2.3. Histological Assessment of Bone Marrow Fibrosis

Bone marrow biopsy samples were fixed in formalin and briefly decalcified before paraffin embedding. Semithin (3 *μ*m-thick) sections were obtained and stained with hematoxylin phloxyn saffron, Masson′s trichrome, and Gordon-Sweet reaction. Myelofibrosis was graded independently by two pathologists. Grading of reticulin fibrosis was according to the quantity and pattern of distribution of reticulin on a scale of 0 to 3, following the recommendations of the European consensus [18]. The category of no fibrosis was termed “MF0”, with scattered linear reticulin with no intersections (cross-overs). The category of mild fibrosis was termed “MF1” with a loose network of reticulin with many intersections, especially in perivascular areas. The category of moderate fibrosis was termed “MF2” with a diffuse and dense increase in reticulin with extensive intersections, occasionally with only focal bundles of collagen and/or focal osteosclerosis. The category of severe fibrosis was termed “MF3” with a diffuse and dense increase in reticulin with extensive intersections with coarse bundles of collagen, often associated with significant osteosclerosis.

## 3. Results

### 3.1. Patient Characteristics

Of the 25 patients reported with a diagnosis of lymphoproliferative disease and BM fibrosis, nine cases were excluded due to the diagnosis of hairy cell leukaemia (*n* = 1), multiple myeloma (*n* = 1), and incomplete medical records (*n* = 7). Thus, a total of 14 cases were finally analyzed. The diagnosis of lymphoma was done on lymph nodes biopsy in 16 patients. The other two patients had lymphoma confined to the bone marrow. There was a clear male predominance with 12 males (75%) and 4 females (25%). Median age at diagnosis was 62 years (range, 16–74). In most cases, clinical characteristics were dominated by B symptoms (*n* = 11, 69%) and splenomegaly (*n* = 8; 50%). Eight patients presented with low-grade lymphoma (follicular lymphoma (*n* = 5), lymphocytic lymphoma/chronic lymphocytic leukemia (*n* = 2), lymphoplasmacytic lymphoma (*n* = 1)). High-grade lymphomas were documented in 5 patients (B-DLCL, *n* = 3; Burkitt lymphoma, *n* = 1, T-cell lymphoblastic lymphoma, *n* = 1) and three patients presented with mantle cell lymphoma. Most of the patients (*n* = 11; 69%) had ECOG scale of less than 2. B-symptoms were found in 11 patients (11/17; 69%) including follicular lymphoma (*n* = 2/5), B-DLCL (*n* = 3/3) mantle cell lymphoma (*n* = 2/3). LDH level was elevated in 10 patients (62%). The IPI score was more than 1 in all patients with B-DLCL and MCL. The FLIPI score was more than 1 in 4/5 pts with follicular lymphoma. Median white blood cell (WBC) was 8.4 k/L (3.0–20.7), hemoglobin level at 12 g/dl (8.4–15), and platelets count at 165 k/L (50–444). Three patients (19%) had low white WBC count, 8 had anemia (50%), and 5 had thrombocytopenia (31%). Myelofibrosis was graded mild in 9 patients, moderate in 6 patients, and severe in one patient. No signs of atypical megakaryocytes or myeloproliferation have been observed. JAK2^V617F^ mutation was negative in the 10 patients analysed.

Patients received a median of 1 treatment line (range, 1–8). One patient had autologous stem cell transplantation (mantle cell lymphoma). Eleven patients (68%) reached complete remission (CR), 4 patients (25%) had had partial remission (PR), and one patient was progressive after the first line of chemotherapy. BM histology was evaluated, at the end of therapy, in 8 patients (CR = 5; PR = 3). MF disappeared in 4 patients (3 patients who were in CR and 1 in PR from lymphoma) and improved in 4 others. Relapse occurred in 8 patients (50%); five of them were in CR at the end of the first line of chemotherapy with disappearance of MF in 3 patients and mild fibrosis in 2 patients; three patients were in PR with only one patient with no fibrosis after the first line of therapy. BM analysis, at relapse, showed mild (*n* = 2) and moderate (*n* = 3) myelofibrosis in all of them.

After a median follow-up of 42 months, 12 (75%) patients were alive, 9 in CR, 2 in PR and 1 with progressive disease. Median overall survival (OS) and disease free survival (DFS) were 72 months, and 61 months respectively.

## 4. Discussion

In addition to primary and postchronic myeloproliferative disorders, myelofibrosis may be associated with a large subset of diseases such as autoimmune disorders or lymphoproliferative diseases. Lymphoid myelofibrosis represents a particular and rare entity in which medullary fibrosis associated with abnormal lymphoproliferation replaces normal hematopoiesis. Hairy cell leukemia is one of the most known lymphoma in which MF is frequently encountered; however, the association with other lymphoproliferation is rarely described. Rare cases have been reported in multiple myeloma, T-cell lymphoma, marginal cell, and lymphoplasmatoid cell lymphoma [3, 4, 12, 16] (Table 2). However, the clinical characteristics and the prognosis of such association are not well known. 

Over a period of seven years, 375 cases were registered, with the diagnosis of lymphoma, in the records of the department of pathology. Twenty-five of them were associated with bone marrow fibrosis which implies a crude incidence of 6.6%. In this study, Myelofibrosis was not specific for a type of lymphoma; however we note in this group of patient a relatively more frequent association of myelofibrosis with low-grade non-Hodgkin lymphoma (8/16; 50%). B-symptoms were frequently present (12/16; 75%), and the LDH level was elevated in 62% of patients at initial presentation. It is important to note that two patients presented with features of idiopathic myelofibrosis without any signs of peripheral lymphoproliferation. The diagnosis of lymphoma in those two patients was done after more than one bone marrow biopsy and immunophenotyping of bone marrow cells. The other clinical and biological parameters were unremarkable (Table 1). MF was mild to moderate in all cases. One patient presented with osteomyelosclerosis and did not respond to chemotherapy. JAK2^V617F^ was negative in the ten cases analyzed. JAK2^V617F^ is found in 50 percent of primary myelofibrosis cases. The observation that none of our lymphoproliferative disease-associated cases were positive for this mutation suggests a distinct etiology from PMF. Response to therapy was satisfactory (CR = 75%; PR = 25%), with a relapse rate, DFS, and OS not different from lymphoma without myelofibrosis. However, the prognostic value of the persistence of MF after chemotherapy cannot be determined in this small group of patient. Cytokines produced by megakaryocytes and monocytes such as PDGF, TGF-*β*, VEGF and *β*-FGF have been shown to play an important role in the development of secondary stromal proliferation, and the TGF-*β* secreted by the tumor cells has been suggested to play an important role in the development of MF conferring a direct relation between lymphoma and MF. In our group of patient, the concomitant regression of MF with the response of lymphoma to chemotherapy and its reappearance with relapse argue for a direct relation between the tumor cell and myelofibrosis.

In conclusion, secondary myelofibrosis to non-Hodgkin′s lymphoma is rare and seems to be more associated with low-grade lymphoma. The clinical course and prognosis are not different from lymphoma without MF. A search for lymphoproliferation should be considered when the cause of MF is not apparent.

## Figures and Tables

**Table 1:** Clinical and biological features of myelofibrosis associated lymphoma patients.

	N (%)
Patients	16
Age (years)	
Median (range)	62 (16–74)
Sex	
Male/female	12/4 (75/25)
Lymphoma histology	
Low grade	8 (50)
MCL	3
DLCL	3
others	2
B symptoms	
Positive	11 (69)
negative	5
Splenomegaly	
yes	8 (50)
no	8 (50)
LDH level (UI/L)	
normal	6 (38)
elevated	10 (62)
WBC count	
median (range) (10^9^/L)	8.4 (3.0-20.7)
Hb level	
median (range) (g/dl)	12 (8.4–15)
platelets count	
median (range) (10^9^/L)	165 (50–444)
Dacryocytes	
Positive/negative/na	1/12/3
Leucoerythrocytic features	
Positive/negative/na	3/10/3
FLIPI	
0–1	1/5 (20)
>1	4/5 (80)
Myelofibrosis grading	
Mild	9 (56)
Moderate	6 (37)
Severe	1
*N*° lines chemotherapy	
1–3 lines	15 (93)
>3 lines	1
Overall response rate	94%
Overall survival (months)	72

DLCL: diffuse large cell lymphoma; FLIPI: follicular lymphoma international prognostic index; LDH: lactate dehydrogenase; MCL: mantle cell lymphoma; WBC: white blood cells; Hb: hemoglobin.

**Table 2:** Reported cases of myelofibrosis and lymphoproliferative disease.

	Sex/Age	Histology	MF degree	B-symptoms	Lymph nodes	SMG	HMG	LDH	Treatment	Response	FU (mo)	Outcome
Takai and Sanada [14]	F/65	T-NHL	marked	+	+	−	−	increased	CHOP	CR	9	A
Kimura et al. [15]	H/55	B-CLL	moderate to severe	na	+	+	+	na	COP, CLB	PR	11	A
Pulsoni et al. [13]	F/58	PCM	extensive	+	−	+	+	na	MP, VMCP-VBAP	PR, relapse	44	D
Orth [12]	F/55	T-NHL	na	na	+	+	+	na	Prednisone, Vincristine	PR	4	A
Meckenstock et al. [10]	H/36	B-NHL	diffuse	+	−	+	+	increased	corticosteroid	—	3	D
Weirich et al. [16]	H/42	T-NHL	marked, diffuse	+	−	+	+	na	CHOP, splenectomy	PR	2	A
Stevenson et al. [8]	F/60	PCM	diffuse	+	−	+	+	na	VAD, melphalan + ASCT	CR	24	A
Kasahara et al. [11]	F/68	B-NHL	marked	+	−	−	−	na	CHOP	< PR	na	D
Abe et al. [19]	F/19	T-NHL	diffuse	na	+	+	+	increased	CHOP	CR	24	A
Uehara et al. [9]	H/69	T-NHL	diffuse	−	+	+	na	increased	na	PR, relapse	30	D
Rao et al. [7]	F/46	T-NHL	marked	+	+	+	+	increased	Splenectomy CHOP and ICE	CR after ICE	11	A
Matsunaga et al. [6]	F/73	B-NHL	4+	+	−	+		increased	Splenectomy, fludarabine, cyclophosph	CR	12	D
Okabe et al. [4]	H/68	T-NHL	diffuse	na	−	+	−	N	Cyclophos, oncovin, prednisone	refractory	9	D
Hatta et al. [20]	H/67	B-NHL	NA	−	+	−	−	increased	na	refractory	14	D
Hagihara et al. [5]	H/68	B-NHL	NA	+	−	+	+	increased	CHOP	CR	na	A

A: alive; CR: complete remission; CLB: chlorambucil; CLL: chronic lymphocytic lymphoma; D: death; HMG: hepatomegaly; ICE: ifosfamide cytarabine etoposide; NHL: non-hodgkin lymphoma; PCM: plasma cell myeloma; na: not available; PR: partial remission; Prog: progressive; SMG: splenomegaly; +: present; −: absent.
